# CCR9-expressing CD14+HLA-DR^hi^ blood monocytes promote intestinal inflammation in IBD

**DOI:** 10.1186/1479-5876-9-S2-P32

**Published:** 2011-11-23

**Authors:** Ludvig Linton, Mats Karlsson, Jeanette Grundström, Per Marits, Annelie Lindberg, Per Karlen, Ola Winqvist, Michael Eberhardson

**Affiliations:** 1 Medicine, Karolinska Institute, Stockholm, Sweden; 2 Clinical Research and Education, Södersjukhuset, Stockholm, Sweden

## Background

Circulating monocytes have been demonstrated to relocate to the intestinal mucosa during intestinal inflammation, but the underlying mechanisms remain poorly understood. Here, we have investigated a subpopulation of blood monocytes expressing high levels of HLA-DR, CCR9 and CCR7 in patients with inflammatory bowel disease (IBD).

## Materials and methods

51 patients with mild-to-severe ulcerative colitis (UC) or Crohn’s disease (CD) were included together with 14 controls. The frequency of CD14^+^HLA-DR^hi^ monocytes was monitored weekly in peripheral blood using flow cytometry. The surface phenotype and cytokine profile of these monocytes were established using flow cytometry and real-time PCR.

## Results

The frequency of CD14^+^HLA-DR^hi^ monocytes was significantly higher in IBD patients with moderate-to-severe disease compared to healthy controls. Furthermore, these monocytes correlated to disease activity in patients with UC and CD. CD14^+^HLA-DR^hi^ monocytes are defined by their high production of pro-inflammatory cytokines and their surface expression of CCR7 and CCR9.

## Conclusions

CD14^+^HLA-DR^hi^ blood monocytes were increased in patients with active inflammatory bowel disease. These monocytes exhibit a pro-inflammatory gut-homing phenotype with regards to their production of inflammatory mediators and expression of CCR9. Our results suggest that these monocytes are important in mediating intestinal inflammation, and provide potential therapeutic targets in IBD.

